# PD-L1 assessment in pediatric rhabdomyosarcoma: a pilot study

**DOI:** 10.1186/s12885-018-4554-8

**Published:** 2018-06-13

**Authors:** Giulia Bertolini, Luca Bergamaschi, Andrea Ferrari, Salvatore L. Renne, Paola Collini, Cecilia Gardelli, Marta Barisella, Giovanni Centonze, Stefano Chiaravalli, Cinzia Paolino, Massimo Milione, Maura Massimino, Michela Casanova, Patrizia Gasparini

**Affiliations:** 1 Department of Research, Tumor Genomics Unit, Genomics Unit, Fondazione IRCCS Istituto Nazionale dei Tumori, via venezian 1, 20133 Milan, Italy; 2 Department of Pediatric Oncology, Fondazione IRCCS Istituto Nazionale dei Tumori, 20133 Milan, Italy; 3 Soft tissues and bone, and pediatric pathology unit, Fondazione IRCCS Istituto Nazionale dei Tumori, 20133 Milan, Italy; 4 Pathology Unit, Fondazione IRCCS Istituto Nazionale dei Tumori, Milan, Italy; 5 Unit of Thoracic Surgery, Fondazione IRCCS Istituto Nazionale dei Tumori, Milan, Italy; 6 Clinical Research Lab (CRAB), Department of Pathology and Laboratory Medicine, Fondazione IRCCS Istituto Nazionale dei Tumori, Milan, Italy

**Keywords:** Pediatric malignancies, Rhabdomyosarcoma, Soft tissue sarcoma, PD-L1 expression, Flow cytometry, Immunohistrochemistry, Primary cell lines

## Abstract

**Background:**

Rhabdomyosarcomas (RMSs) are the most frequent soft tissue sarcoma in children and adolescents, defined by skeletal muscle differentiation and the status of FOXO1 fusions. In pediatric malignancies, in particular RMS, scant and controversial observations are reported about PD-L1 expression as a putative biomarker and few immune checkpoint clinical trials are conducted.

**Methods:**

PD-L1 assessment was evaluated by immunohistochemistry (IHC) utilizing two anti-PDL1 antibodies, in a pilot cohort of 25 RMS. Results were confirmed in primary and commercial RMS cell lines by cytofluorimetric analysis and IHC.

**Results:**

PD-L1 expression was detectable, by both anti-PD-L1 antibodies, in the immune contexture of immune cells infiltrating and/or surrounding the tumor, in 15/25 (60%) RMS, while absent expression was observed in neoplastic cells. Flow cytometry analysis and PD-L1 IHC of commercial and primary RMS cell lines confirmed a very small percentage of PD-L1 positive-tumor cells, under the detection limits of conventional IHC. Interestingly, increased PD-L1 expression was observed in the immune contexture of 4 RMS cases post chemotherapy compared to their matched pre-treatment samples.

**Conclusion:**

Here we identify a peculiar pattern of PD-L1 expression in our RMS series with scanty positive-tumor cells detected by flow cytometry, and recurrent expression in the immune cells surrounding or infiltrating the tumor burden.

**Electronic supplementary material:**

The online version of this article (10.1186/s12885-018-4554-8) contains supplementary material, which is available to authorized users.

## Background

Rhabdomyosarcoma (RMS) is a highly aggressive tumor arising from immature mesenchymal cells committed to skeletal muscle differentiation, it represents the most frequent soft tissue sarcoma in childhood. Although it is generally responsive to the multimodal therapeutic approaches including intensive chemotherapy, the prognosis of RMS depends on several different variables and for some patients the outcome remains dismal [1].

Pediatric RMS has two major histological subtypes, each with distinct clinical, molecular, and genetic features: the embryonal RMS (ERMS) are more frequent (~ 80% of cases) with a higher incidence in younger children; and the alveolar RMS (ARMS), less frequent (~ 20% of cases) but more aggressive and often resistant to conventional chemo- and radiotherapy, resulting in a 5-year survival rate of only 30% [2–5]. Specifically, patients with alveolar histology continue to have less than optimal outcome, and most patients with distant metastasis or relapsing disease do not achieve long term cure [6]. Furthermore, long-term survivors may endure from important late functional sequelae related to the burden of multimodal therapies they received. Therefore, the identification and development of more efficient and less toxic therapeutic approaches is absolutely needed [7].

PD-1, a type I transmembrane glycoprotein cell surface receptor expressed on T- and pro-B cells, is an immunoreceptor belonging to the CD28/CTLA-4 family of T-cell regulators and, functioning as an immune checkpoint, plays a critical role in downregulating the immune system by preventing the activation of T-cells. PD-1 binds to two ligands, PD-L1 and PD-L2, which block PD1 receptor e induce PD-1 signaling and T-cell ‘exhaustion’. Recently, targeting the PD-1/PD-L1 immune checkpoint pathway has proved to improve adults patients’ survival, but with less toxicity than conventional treatments, possibly stimulating the anti-tumor immunity by activating the patients’ own immune system [7]. Encouraging clinical benefits of PD-1/PD-L1 checkpoint blockade have been demonstrated in over 15 different malignancies, among which melanoma [8–10], lung cancer [11, 12], genitourinary tract cancer [13], Hodgkin lymphoma [13] and sarcoma [14] confirming that host immune responses are essential in most neoplasms [15]. As the PD-1 pathway may be a key mechanism of immune escape in a subgroup of patients in several malignancies, PD-L1 expression in tumor or inflammatory cells is a candidate biomarker [12]. However, the only limitation is that PD-L1 status is not effective in identifying the fraction of PD-L1 negative patients that may also benefit from immune therapy [7, 16].

For pediatric malignancies, only a few anti-PD-1 and anti-PD-L1 clinical trials are ongoing and little is known regarding the prognostic and predictive implications of PD-L1 in childhood tumors, in particular RMS. Moreover, to our knowledge, no responses have been reported to anti-PD1 or anti-PD-L1 as a single drug in RMS. Thereby, in an attempt to further describe the immune environment of RMS, we evaluated PD-L1 expression, in a cohort of 25 RMS specimens utilizing two anti-PD-L1 antibodies by immunohistochemistry (IHC) approach on Formalin-Fixed Paraffin Embedded (FFPE) RMS tissues and cytoblocks from RMS cell lines. Our observations were further confirmed by flow cytometry analysis in RMS cell lines, both commercial and primary cultures derived from surgical RMS specimens.

## Methods

### Patients and tissue samples

This study was conducted on a retrospective cohort of patients who were pathologically diagnosed with RMS at Fondazione IRCCS Istituto Nazionale dei Tumori, Milano. Twenty-five FFPE RMS tissues were retrieved from the archives of the Department of Diagnostic Pathology and Laboratory Medicine of our Institute and were available for evaluation of PD-L1 expression by IHC analysis.

The clinical pathologic variables such as sex, age, tumor size, histology, IRS, site of onset, stage, and follow up information were assessed and reviewed. The study was approved by the Internal Review Board and the Ethics Committee of the institutions (*CE N. INT 133–16)*. All patients’ parents or their guardians gave their written informed consent for diagnosis and research activities when they were admitted to the hospital. All cases were assessed for the presence of PAX3/7-FOXO1 fusion transcript. In all cases FFPE material was available for reclassification, following the updated WHO criteria for soft tissue sarcomas (2013) into ERMS and ARMS by expert pediatric sarcoma pathologists (SLR, PC, MB).

### Immunohistochemistry

PD-L1 protein expression, together with several antibodies specific for the immune infiltrate component, was investigated by IHC methods on consecutive slides from FFPE RMS tumor samples. Specifically for PD-L1 two different antibodies were considered: clone anti PD-L1-CD274, SP142 (Roche Diagnostic, USA), and clone anti PD-L1 22C3 (Dako, Glostrup, Denmark). Briefly, 2.5/3 μm-thick were cut from paraffin blocks, dried, de-waxed, rehydrated; in particular slides were unmasked with Dako PT-link, EnVision™ FLEX Target Retrieval Solution (Dako, Glostrup, Denmark) High pH, 96 °C - 30 min for PD-L1-CD274, (SP142, dilution 1:100), and Low pH, 98 °C – 30 min for PD-L1 clone 22C3 (Dako, Denmark, diluition 1:50). Finally, both antibody were incubated with a commercially available detection kit (EnVision™ FLEX+, Dako, Denmark) in an automated Immunostainer (Dako Autostainer System). A FFPE H460 cell line xenograft was utilized as a positive control for PD-L1 marked expression within tumor cells.

In addition to PD-L1, the following antibodies were utilized to characterize the immune infiltrate component: CD3 (anti-T cells, Dako), CD68 (anti-macrophage and monocyte, Dako), CD20 (anti-B cells, Dako), CD163 (anti-macrophage, Novocastra), CD56 (anti-T and natural killer cells, Dako), and CD57 (anti-T and natural killer cells, Dako). Antibodies were utilized with the following dilution: PD-L1 (1:100), CD3 (1:100), CD20 (1:400), CD68 (1:3000), CD163 (1:200), CD57 (1:100), CD56 (1:400).

### Interpretation of PD-L1 expression by immunohistochemistry

For both antibodies, PD-L1 staining was evaluated in tumor cells (TC) and in non-neoplastic cells enclosed in stromal microenvironment, named tumor infiltrating cells (IC) by two experienced pathologists (SLR, PC, MB). According to PD-L1 antibodies manufacturer’s, we distinguished different classes of staining and assigned scores following the summarized table:

**Table:** 

TC score	TC definition	IC score	IC definition
TC 0	< 1%	IC 0	< 1%
TC 1	1% > 5%	IC 1	1 > 5%
TC 2	5% < 50%	IC 2	5 < 10%
TC 3	≥50%	IC 3	≥10%

### Cell lines

A panel of cell lines was analyzed by flow cytometry analysis to verify the presence of PD-L1 protein expression in an in vitro model composed of merely tumor RMS cells. RH30 (ARMS) and NCI-H460 (large cell lung cancer) cell lines were obtained from the American Type Culture Collection (ATCC) and grown according to guidelines. On the other hand, five primary RMS cell cultures were established from fresh tumor specimens by mechanical and collagenase II enzymatic dissociation, followed by culturing and propagating in Amniomax-C100 medium (Invitrogen), and characterized for the PAX3/7-FOXO1 translocation by FISH. For each cell line, cell blocks were also prepared and utilized for IHC analysis of PD-L1.

### Flow cytometry analysis

For cell staining, single cell suspensions of all 7 cell lines were washed and incubated in staining buffer (PBS 1× containing 1% BSA and 2 mM EDTA) with anti-PDL-1 (clone B7-H1, E-bioscience) and appropriate IgG Isoytype control, all diluted 1:10 for 30 min at 4 °C cells. Prior to acquisition, samples were incubated with 7-AAD viability staining solution (10 μl/tube) for exclusion of dead cells. Flow cytometry data were acquired using FACSCalibur cytometer (Becton Dickinson) and analyzed by Flowjo software.

## Results

### Clinicopathological features

The retrospective cohort comprised 25 RMS (13 ERMS, 11 ARMS, and 1 RMS with sclerosing features) tissues. Our series included ERMS and ARMS (fusion transcript positive and negative) either collected at diagnosis (pre-treated and not), at time of progression of disease, or during therapy, so to recapitulate different disease courses of RMS malignancy. Clinical and pathological characteristics are summarized in.

### PD-L1 protein status and characterization of immune contexture by IHC

To evaluate PD-L1 protein expression in RMS and characterize the immune cells present within each specimen, we performed a thorough IHC analysis in our cohort of 25 RMS histological samples. Two different anti PD-L1 antibodies were utilized and for both PD-L1 staining was evaluated and scored in tumor cells (TC) and in non-neoplastic cells enclosed in stromal microenvironment, named tumor infiltrating cells (IC) for each RMS specimen (Table 1).

**Table 1:** PD-L1 expression

ID	Histotype	Fusion Trascript	PD-L1 IHC				Comments	Status	T	N	M	IRS	Outcome
			TC score		IC score								
			Clone SP142	Clone 22C3	Clone SP142	Clone 22C3							
RMS1	ERMS	negative	0	0	2	2	IC only outside tumor	In therapy	T2B	N0	M1	4	DOD
RMS2	ARMS	PAX7-FOXO1	0	0	0	0		Pre-treated	T1	N0	M0	1	DOD
RMS3	ERMS	negative	0	0	0	0		In therapy	T2B	N0	M1	4	DOD
RMS4	ARMS	negative	0	0	0	0		At diagnosis	T2B	N0	M1	4	DOD
RMS5	ERMS	negative	0	0	0	0		At diagnosis	T2A	N0	M1	4	1°RC
RMS6	ARMS	negative	0	0	3	3	IC outside and infiltrating tumor	At diagnosis	T2A	N1	M1	4	DOD
RMS7	ARMS	PAX3-FOXO1	0	0	1	1	Few cells in IC outside and infiltrating tumor	At diagnosis	T1A	N0	M1	4	DOD
RMS8	ERMS	negative	0	0	2	2	IC only outside tumor	Pre-treated	T2B	N0	M0	3	1°RC
RMS9	ERMS	negative	0	0	3	3	IC outside and infiltrating tumor	At diagnosis	T2B	N0	M0	3	1°RC
RMS10	ARMS	negative	0	0	2	2	IC only outside tumor	Pre-treated	T2B	N0	M0	3	2°RC
RMS11	RMS	negative	0	0	0	0		At diagnosis	T2B	N1	M0	3	DOD
RMS12	ARMS	PAX7-FOXO1	0	0	2	2	IC only outside tumor	At diagnosis	T2B	N1	M0	3	1°RC
RMS13	ARMS	PAX7-FOXO1	0	0	3	3	IC outside and infiltrating tumor	Pre-treated	T2A	N0	M1	3	DOD
RMS14	ARMS	PAX3-FOXO1	0	0	0	0		At diagnosis	T1A	N0	M1	4	DOD
RMS15	ERMS	negative	0	0	2	2	IC only infiltrating tumor	At diagnosis	T1A	N0	M0	3	1°RC
RMS16	ARMS	PAX3-FOXO1	0	0	3	3	IC outside and infiltrating tumor	At diagnosis	T2B	N1	M0	3	1°RC
RMS17	ARMS	PAX3-FOXO1	0	0	0	0		At diagnosis	T2B	N1	M1	4	DOD
RMS18	ERMS	negative	0	0	0	0		At diagnosis	T2B	N0	M0	3	1°RC
RMS19	ERMS	negative	0	0	2	2	IC outside and infiltrating tumor	post-treatment	T2B	N0	M0	3	1°RC
RMS20	ARMS	negative	0	0	0	0		post treated	T1A	N0	M0	3	1°RC
RMS21	ERMS	negative	0	0	2	2	IC outside and infiltrating tumor	post-treatment	T2B	N1	M0	3	DOD
RMS22	ERMS	negative	0	0	2	2	IC outside and infiltrating tumor	At diagnosis	T2B	N0	M0	3	1°RC
RMS23	ERMS	negative	0	0	3	3	IC outside and infiltrating tumor	post-treatment	T2B	N0	M0	3	1°RC
RMS24	ERMS	negative	0	0	0	0		At diagnosis	T2B	N0	M0	3	1°RC
RMS25	ERMS	negative	0	0	2	2	IC outside and infiltrating tumor	post-treatment	T2B	N0	M0	3	1°RC

**Fig. 1:**
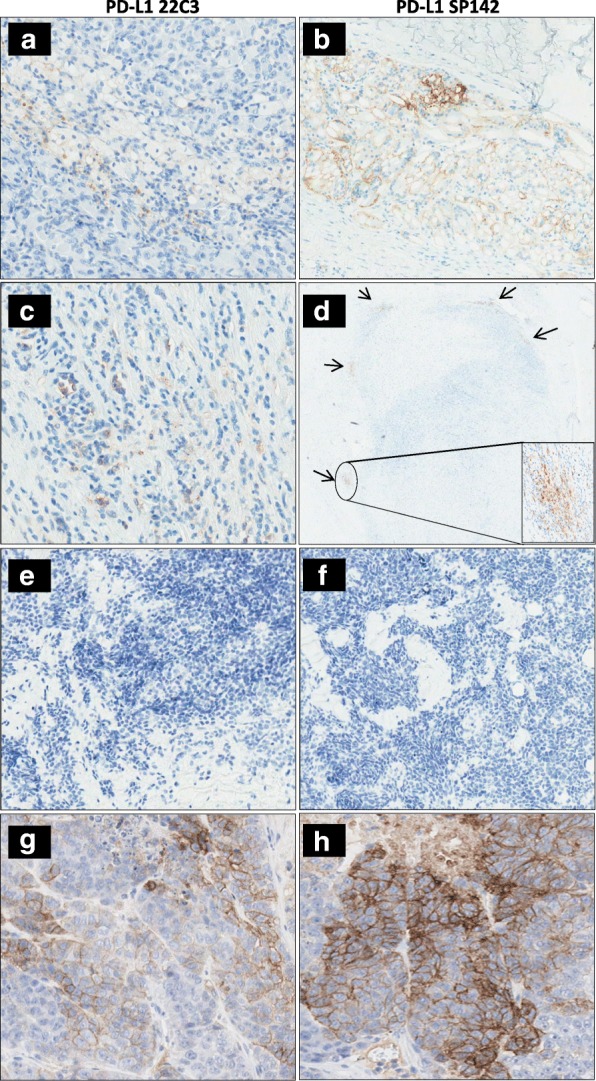
Expression pattern of PD-L1 in RMS. Comparable IHC results were obtained by PD-L1 clones SP124 and 22C3. (**a** and **b**) RMS16 showing a marked and consistent PD-L1-staining in the immune component IC3: staining visualized in areas surrounding and infiltrating the tumor burden. (**c** and **d**) RMS10 displaying a weak and focal staining of PD-L1, IC2, uniquely in the immune component encircling the tumor (as pointed out by the arrows). (**e** and **f**) An absent PD-L1 expression in all compartments, TC0 IC0: tumor and infiltrating immuno-component (RMS7). (**g** and **h**) H460, utilized as a positive control, reveals a marked expression in the tumor cells

IHC results were comparable for both PD-L1 antibodies, though immunostaining with clone 22C3 proved to be a bit more fainted. Overall, PD-L1 expression for both antibodies resulted completely absent in tumor cells (TC0) of our entire cohort. Interestingly, PD-L1 expression was observed in the immune contexture (IC1-IC3) in 15/25 (60%; 6/11 ARMS, 9/14 ERMS) RMS evaluated. Of the 15 RMS displaying PDL1 expression 5 RMS scored IC3, 8 RMS had IC2 and only one RMS showing occasional PD-L1 expression in single immune cells outside the tumor burden (IC1). We were also able to describe a peculiar staining pattern for the PDL1-expressed RMS: a marked and continuous protein expression in both the immune cells infiltrating and surrounding the tumor (Fig. 1a and b) was observed in 10 RMS and a moderate, nest-like, focal and not diffuse pattern of PD-L1 protein expression exclusively in the immune cells surrounding the tumor burden (Fig. 1c and d) was reported in 4 RMS. Only one specimen revealed PD-L1 expression merely in the infiltrating immune cells in the tumor burden. At last, PD-L1 expression was absent both in the tumor cells (TC0) and in the immune component (IC0) in 10/25 (40%) RMS specimens (Fig. 1e and f). As opposed to our positive control (H460) that clearly expressed PD-L1 in tumor cells, none of our 25 RMS series revealed expression in tumor cells (Fig. 1g and h).

**Fig. 2:**
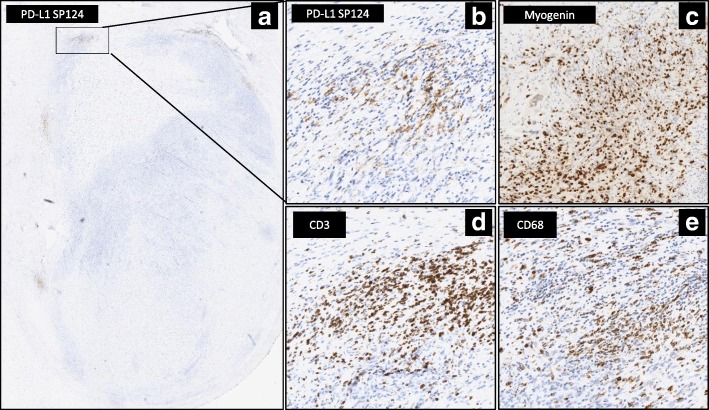
Characterization of infiltrating immune contexture. (**a**) RMS10 with a PD-L1 scoring of TC0 and IC2 in the areas surrounding the tumor with a faint staining and in a focal pattern (as pointed out by the arrows). (**b**-**e**) Magnification (40X) of the area within the box, displaying different IHC expression staining: (**b**) PD-L1(Clone 1SP124) expression in the immune contexture and not in tumor cells, (**c**) Myogenin positive expression of neoplastic cells, (**d**) CD3 revealing T-lymphocytes positivity, (**e**) and CD68 staining confirming macrophages positivity

Moreover, to better define PD-L1 protein expression in the immune component (infiltrating or surrounding the tumor), a panel of linage specific antibodies (CD3, CD68, CD20, CD163 CD56, CD57) was utilized, on samples with available material (Additional file 1: Table S2). Our observations revealed that PD-L1 staining co-localized with area showing marked positivity for CD3+ T-lymphocytes and CD68+ macrophages, while lack of co-localization with tumor cells is highlighted by nuclear staining of myogenin in RMS tissue (Fig. 2a-e).

Although no statistical analysis was possible due to the limited size and the heterogeneity of the cohort, it can be observed that PD-L1 staining in the IC does not seem to correlate with neither the fusion transcripts status, the outcome nor any other clinical feature.

### PD-L1 expression by flow cytometry in cell lines

In order to confirm absence of PD-L1 staining by IHC in the neoplastic cells, we evaluated the protein expression by flow cytometry in cancer cell lines. A total of 6 RMS cell lines were available for FACS analysis: two commercial cell line (RH30 and RD) and 5 primary cell cultures established from surgical tumor specimens whose corresponding histological samples were also analyzed by IHC (RMS12-RMS16). All results are summarized in Table 2.

**Table 2:** PD-L1 expression in FFPE RMS tissues, derived cell lines and cytoblocks

ID #	Cell line	Fusion Trascript	PD-L1 (IHC)		Flow Cytometry	PD-L1 (IHC)
			FFPE tissue		DERIVED CELL LINE	CYTOBLOCK (cell line)
			TC score	IC score	% TC	
RMS12	RMS-GD	PAX7-FOXO1	0	2	2,10%	0
RMS13	RMS-ME	PAX7-FOXO1	0	3	1,93%	0
RMS14	RMS-GJ	PAX3-FOXO1	0	0	5,55%	0
RMS15	RMS-SG	negative	0	2	2,83%	0
RMS16	RMS-BI	PAX3-FOXO1	0	3	12,25%	1–5 TC

**Fig. 3:**
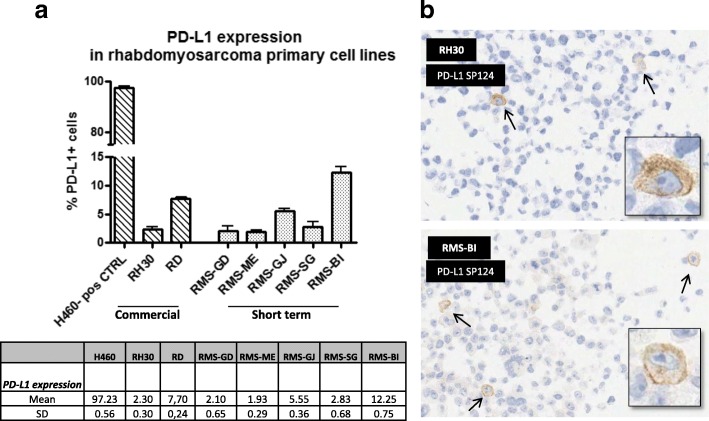
PD-L1 expression in RMS cell lines. **a** Expression of PD-L1 by cytofluorimetric analysis of 2 RMS commercial cell lines (RH30 and RD), 5 short term RMS cultures (RMS-GD, RMS-ME, RMS-GJ, RMS-SG, RMS-BI) and H460 lung cell line utilized as control. **b** PDL1 IHC of RMS short term cultures’ cytoblocks showing a few (1–8) PD-L1 stained cells (enlarged in the box)

Utilizing a lung cancer cell line H460 as a positive control for PD-L1 positivity (97% positive for PD-L1), all RMS cell lines exhibited a relatively low expression ranging from 2 to 12% (average of PD-L1 positive cells; Fig. 3a).

Cytoblocks derived from RMS cell lines were prepared and assessed for expression of PD-L1 in tumor cells by IHC to verify the correlation between results obtained by flow cytometry and IHC methodologies. Indeed, PD-L1 staining was observed in very few tumor cells (1–8 cells/slide) of RH30 and primary cell line RMS-BI (Fig. 3b).

### Dynamic changes of PD-L1 expression induced by chemotherapy

**Fig. 4:**
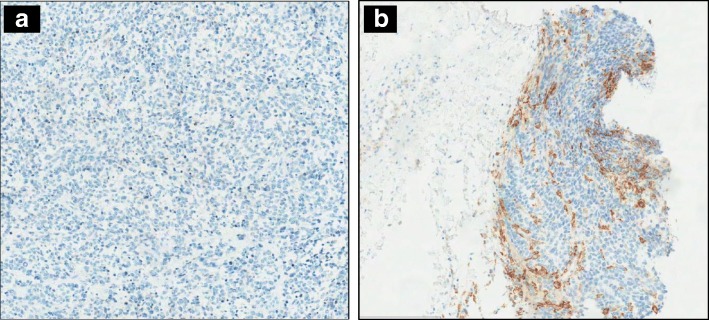
Changes of PD-L1 protein expression. PD-L1 (ab PD-L1 SP124) expression is observed in the same patient but monitored at different time lapse: (**a**) RMS2 (at first progression, pretreated with radiotherapy and chemotherapy) is completely lacking PD-L1 expression (TC0,IC0); (**b**) while RMS13 (at second progression and post several lines of treatments) display a consistent pattern of staining (TC0, IC3) by the immune cells present outside and inside the tumor burden

Among our cohort, 8 RMS tissues from 4 RMS patients were evaluated at different tumor progression time. Specifically, RMS2 and RMS13 (ARMS) represented the first progression of disease and its corresponding relapse sample. Moreover, RMS18, RMS22 and RMS24 were all ERMS tissues obtained at diagnosis, while RMS19, RMS23 and RMS25 were their corresponding tumor progression post several lines of treatment (possible cycle treatments: Vincristine and Irinotecan, Vinorelbine and Endoxan, Ifosfamide/Doxorubicina/Actinomicina and Ciclofosfamide/ Doxorubicina/ Vincristina). Interestingly, PDL1 expression was reported as negative in RMS2 (Fig. 4), RMS24, RMS18 (Additional file 2: Figure S1A and C) or a weakly expressed in RMS22 (Additional file 2: Figure S1E) in samples collected at diagnosis and first tumor progression, but it revealed to show a marked PD-L1 staining in the immune component both surrounding and infiltrating the tumor burden in the corresponding tissue obtained post therapy (Fig. 4 and Additional file 2**:** Figure S1B,D,F). Our results strongly suggest that damages and selective pressure caused by chemotherapy can reactivate a tumor immune response.

## Discussion

Successful results obtained with pembrolizumab and nivolumab in melanoma, NSCLC, sarcoma and other malignancies [10–12, 17, 18], have been recently reported, bringing forward immune checkpoint inhibitors as potential treatment options. Since there has been limited research to investigate the clinical and prognostic significance of the PD-1/PD-L1 axis in pediatric malignancies, in the present study we assess the presence of PD-L1 expression in pediatric RMS primary tumors and corresponding cell lines. Our results identify PD-L1 expression in 60% of RMS analyzed, detecting mild/moderate staining uniquely in the immune cells surrounding the tumor burden and/or in those infiltrating the tumor, thus never observing expression in the neoplastic cells.

Current studies have pointed out that high expression of PD-L1 in tumor cells is associated with poor prognosis in NSCLC [11, 12, 19], ovarian cancer [20] and kidney cancer [21], melanoma [22], renal cancer [21], Hodgkin lymphoma [16] and bone and soft tissue sarcoma [15, 23, 24]. Our study demonstrated, by using two different anti-PD-L1 clones that PD-L1 expression is confined in immune cells infiltrating and/or surrounding the tumor burden, but not in RMS tumor cells. To further confirm our results, we assessed PD-L1 expression of RMS cell lines by two different techniques (flow cytometry and IHC on cytoblocks) and using two different clones of anti-PD-L1 antibodies: we were able to detect a very low percentage of tumor cells by flow cytometry, due to the high sensitivity of this technique, that accordingly was hardly detectable with a conventional IHC approach on FFPE cytoblocks. This data confirms the almost completely absence of PD-L1 expression in RMS tumor cells, even when different techniques and antibody clones were utilized. Low expression of PD-L1 in RMS was also reported by Torabi et al. detecting positivity in 3/96 cases in a TMA [24]. Our data are in contrast with those reported by Kim et al. showing an expression of PD-L1 in tumor cells in 37% (12/32) of specimens [15]. However, discordant observations in expression of PD-L1 could be explained by use of different anti-PD-L1 antibodies, staining procedures, and antigen retrieval techniques.

Interestingly, an important subdivision of anticancer immunity in humans into three main phenotypes is represented by Chen & Mellman: immune-desert, immune-excluded and immune-inflamed. Accordingly to this classification, our observations enabled us to subdivide our cohort in 4 ‘immune-inflamed’ RMS, displaying expression of PDL1 in immune cells surrounding and within the tumor burden, which may likely respond to anti-PD-L1/PD-1 therapy, and 5 ‘immune-excluded’ RMS with PD-L1 staining present in immune cells that do not penetrate the parenchyma of the tumor but rather are retained in the surrounding stroma, which are expected to rarely respond to PD-L1/PD-1 agents [25]. At last, 7 specimen were revealed to be ‘immune-desert’ and characterized by very few T-cells in either the parenchyma or the stroma of the tumor burden, therefore not responsive to PD-L1/PD-1 agents [25]*.*

Dynamic changes of PD-L1 protein expression were observed in 4 RMS tumors evaluated at diagnoses/first progression of disease and at a (second) tumor progression post-treatment, suggesting that damages induced by chemotherapy treatments in tumor cells and stroma may foster an inflammatory microenvironment and recruitment of PD-L1 expressing immune cells, creating an immune contexture possibly druggable by immune checkpoint inhibitors. Indeed randomized clinical trial using immunotherapy in second line after chemotherapeutic treatments in NSCLC indicated successful response rate [12, 26]. These results also reinforce the concept to perform biopsies, when possible, prior of initiating an immune checkpoint treatment to confirm the immune contexture status at that particular time, as it is easily influenced and prone to changes [even if the biomarker validity of PD-L1 positivity is highly debatable].

In view of our results, the lack of expression by the neoplastic cells discourages us to consider RMS an immunogenic tumor possibly explaining the lack of literature of immune checkpoint inhibitors in pediatric RMS. However, the distinct PD-L1 expression pattern observed in our cohort, could be critical in discriminating ‘immune-inflamed’ from ‘immune-excluded’ specimen, making a significant difference in discriminating those patients that may benefit from PD-1/PD-L1 therapy.

## Conclusion

To the date, clinical trials with immune checkpoint inhibitors in pediatric malignancies are few and with mainly unsatisfactory results, thus an effort to characterize the immune contexture is needed. In this study, we demonstrate by different techniques and in multiples setting the low expression of PDL1 by RMS, and we observed a possible complementary role of chemotherapy as igniter of ‘inflamed tumors’. Taken altogether these data may suggest the possibility of a combination with conventional chemotherapy and PD-L1 checkpoint blockade.

## Additional files

Additional file 1: **Table S1.** Clinico-pathological features. A summary of all the clinic-pathological features of the analyzed cohort. **Table S2.** Characterization of the immune infiltrate contexture. This table display the results of the IHC performed, only on RMS with abundant FFPE material, to characterized the immune contexture of RMS. (ODP 30 kb)

Additional file 2: **Figure S1** PD-L1 expression pre and post therapy. Changes in PD-L1 expression are revealed in pre- and post-treatment RMS tissue from the same patients: RMS24, RMS18, RMS22 (A,C and E), all at diagnosis and with no prior treatment, show absence or a mild expression of PD-L1 in the immune component; RMS25, RMS19, RMS23 (B,D and F), all following several lines of treatments, mainly chemotherapy, display a moderate expression in the immune contexture outside and infiltrating the tumor burden. (PPTX 6110 kb)

## Abbreviations

ARMS: Alveolar RMS
ERMS: Embryonal RMS
FFPE: Formalin-Fixed Paraffin Embedded
FISH: Fluorescent in situ hybridization
IC: Infiltrating cells
IHC: Immunohistochemistry
NSCLC: Non-small cell lung cancer
RMS: Rhabdomyosarcoma
TC: Tumor cells
