# Supplementary figures and images for: PD-L1 assessment in pediatric rhabdomyosarcoma: a pilot study
Source: BMC Cancer. 2018 Jun 13;18:652. doi: 10.1186/s12885-018-4554-8 (PMC6001160; doi:10.1186/s12885-018-4554-8)

## Slide 1
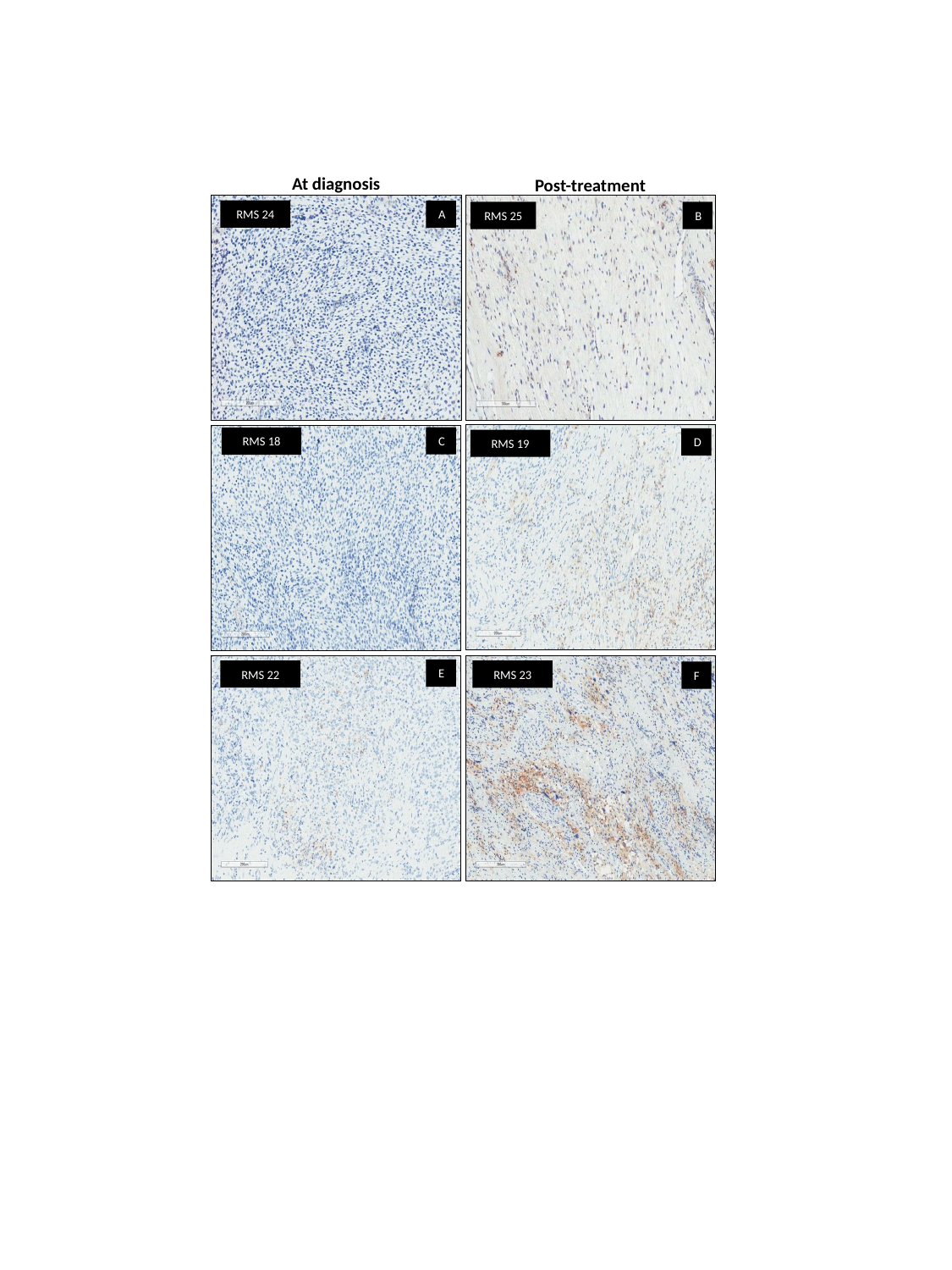

At diagnosis
Post-treatment
RMS 24
A
RMS 25
B
C
RMS 18
D
RMS 19
E
RMS 22
RMS 23
F

Supplement: Supplementary file 2 — Figure S1 PD-L1 expression pre and post therapy. Changes in PD-L1 expression are revealed in pre- and post-treatment RMS tissue from the same patients: RMS24, RMS18, RMS22 (A,C and E), all at diagnosis and with no prior treatment, show absence or a mild expression of PD-L1 in the immune component; RMS25, RMS19, RMS23 (B,D and F), all following several lines of treatments, mainly chemotherapy, display a moderate expression in the immune contexture outside and infiltrating the tumor burden. (PPTX 6110 kb) [file 12885_2018_4554_MOESM2_ESM.pptx]
